# No evidence of Alzheimer’s disease pathology in mice infected with *Toxocara canis*

**DOI:** 10.1051/parasite/2025019

**Published:** 2025-04-09

**Authors:** Ondřej Vosála, Barbora Šmídová, Jan Novák, Jan Svoboda, Tomáš Petrásek, Iveta Vojtěchová, Tomáš Macháček

**Affiliations:** 1 Department of Parasitology, Faculty of Science, Charles University Viničná 7 Prague 2 12844 Czechia; 2 Department of Biochemical Sciences, Faculty of Pharmacy in Hradec Králové, Charles University Akademika Heyrovského 1203 Hradec Králové 50005 Czechia; 3 Institute of Medical Microbiology, First Faculty of Medicine, Charles University and General University Hospital in Prague Studničkova 7 Prague 2 12800 Czechia; 4 Laboratory of Neurophysiology of Memory, Institute of Physiology, Czech Academy of Sciences Vídeňská 1083 Prague 4 14200 Czechia; 5 Sleep and Chronobiology Research Centre, National Institute of Mental Health Topolová 748 Klecany 25067 Czechia

**Keywords:** *Toxocara canis*, Neurotoxocarosis, Alzheimer’s disease, Amyloid-β, Infectious hypothesis

## Abstract

The potential link between the infections and the development of Alzheimer’s disease (AD) has led to speculations about the role of various pathogens in triggering amyloid-β (Aβ) overproduction, possibly leading to AD onset. The globally distributed dog roundworm *Toxocara canis* was suggested to be a suitable candidate due to neurotropism of the larvae and infection chronicity. This study investigated whether chronic *T. canis* infection induces AD-like pathology in mice and whether Aβ is toxic to *T. canis*. BALB/c and APP/PS1 transgenic mice, which overproduce Aβ, were infected with *T. canis* L3 larvae and monitored for larval burden, Aβ accumulation, and behavioral changes. *In vitro* tests of recombinant Aβ toxicity against the larvae were also performed. Despite the presence of *T. canis* larvae in the central nervous system 8 and 16 weeks post-infection, no significant increase in Aβ concentration or AD-related behavioral alterations were observed. Aβ was detected on the surface and within the intestines of *T. canis* larvae, but *in vitro* exposure to recombinant Aβ did not affect larval viability or morphology. Our findings suggest that *T. canis* infection does not trigger AD-like pathology in mice, and Aβ does not act as an antiparasitic agent. This challenges the emerging hypothesis that chronic neurotoxocarosis infections may contribute to AD development.

## Abstract (fr)

Le lien potentiel entre les infections et le développement de la maladie d’Alzheimer (MA) a suscité des spéculations sur le rôle de divers agents pathogènes dans le déclenchement de la surproduction de β-amyloïde (βA), pouvant conduire à l’apparition de la MA. *Toxocara canis*, un nématode du chien, répandu mondialement, a été suggéré comme un candidat potentiel en raison du neurotropisme de ses larves et de la chronicité de son infection. Cette étude examine si une infection chronique à *T. canis* induit une pathologie de type MA chez la souris et si la βA est toxique pour *T. canis*. Des souris transgéniques BALB/c et APP/PS1, qui surproduisent la βA, ont été infectées par des larves L3 de *T. canis* et la charge larvaire, l’accumulation de βA et les changements comportementaux ont été étudiés. Des tests *in vitro* de toxicité de la βA recombinante contre les larves ont également été réalisés. Malgré la présence de larves de *T. canis* dans le système nerveux central 8 et 16 semaines après l’infection, aucune augmentation significative de la concentration de la βA ni d’altération comportementale liée à la MA n’ont été observées. La βA a été détectée à la surface et dans les intestins des larves de *T. canis*, mais l’exposition *in vitro* à la βA recombinante n’a pas affecté la viabilité ou la morphologie des larves. Nos résultats suggèrent que l’infection à *T. canis* ne déclenche pas de pathologie de type MA chez la souris, et que la βA n’agit pas comme agent antiparasitaire. Cela remet en cause l’hypothèse émergente selon laquelle les infections chroniques par neurotoxocarose pourraient contribuer au développement de la MA.

## Introduction

The dog roundworm *Toxocara canis* (Werner, 1782) (Nematoda: Toxocaridae) is commonly described as a single-host intestinal parasite of canids [42, 51]. However, this parasite utilizes a broad spectrum of species, including humans [7, 51], as paratenic hosts. In them, L3 larvae undergo somatic migration, targeting predominantly the central nervous system (CNS). While *T. cati* seems to accumulate in the cerebellum, *T. canis* prefers the hemispheres [8, 22]. In mice, the first L3 larvae appear in the CNS as soon as 1 week post-infection (WPI) [41], but likely as a consequence of brain immune milieu modulation [36, 46], they remain viable up to several years [1]. Their traumatic migration through the CNS parenchyma is marked by macroscopic surface hemorrhage formation [22]. Moreover, typical hallmarks of neural destruction are observed: demyelination [40], which could be correlated with behavioral changes [23, 37], and axonal damage as indicated by the increase in amyloid-β precursor protein (AβPP) [40, 45].

AβPP can undergo a process called amyloidogenesis, resulting in the production of amyloid-β (Aβ). This small peptide is most often associated with being the main building block of “senile plaques” [50], an infamous pathological hallmark of Alzheimer’s disease (AD). However, Aβ still has some physiological functions [30, 35], including the proposed role of antimicrobial peptide in CNS innate immunity response [20, 44]. Given this role, an infectious hypothesis (IH) of AD, connecting physiological overproduction due to neuroinfection with pathological neurocytotoxicity of Aβ, was formed. IH was initially based on prion research data [49], which were later accompanied by virological studies [3, 4, 13, 26, 48]. Although *in vitro* and *in vivo* data collected from experimentally infected animals suggest that viral presence in CNS might increase Aβ production, the robust and conclusive connection between infections and AD development has not been described so far [2, 16, 28, 53]. Apart from viruses, both prokaryotic and eukaryotic unicellular pathogens were also investigated as potential triggers of Aβ overproduction, possibly leading to AD symptomatology. Importantly, increased levels of Aβ were detected in mouse brains infected by *Porphyromonas gingivalis* or *Candida albicans* [15, 52]. However, neither experiments with pathogenic bacteria [33, 38, 47], yeasts [39, 52], or even protozoan *Toxoplasma gondii* [9, 43] brought irrefutable evidence to credit IH.

As for neurotropic helminths, it was speculated that *T. canis* could be associated with the development of neurodegenerative disorders, including AD [18], mainly due to the chronic nature of the infections and worldwide distribution. Notably, *in vivo* data indicated increased production of AβPP [10, 40] and Aβ [10] resulting from chronic (14–20 WPI) neurotoxocarosis in mice. However, the data are scarce and lack more complex insight, which would enable a better understanding of the link between *T. canis* infection, Aβ production, and AD symptomatology. Here, we investigated whether the chronic infection of mice with *T. canis* triggers increased Aβ production and AD-related behavioral changes. Additionally, the effects of Aβ on *T. canis* larvae both *in vitro* and *in vivo* were assessed to test its potential antimicrobial properties.

## Materials and methods

### Ethics statement

Animal experiments were performed following European and Czech legislation (EU Directive 2010/63/EU, Act No 246/1992). The project was approved by the animal welfare committees of the Faculty of Science, Charles University, and the Ministry of Education, Youth and Sports of the Czech Republic (MSMT-1573/2022-5). Intraperitoneal ketamine (250 mg/kg) and xylazine (25 mg/kg) injection and subsequent exsanguination by transcardial perfusion were combined to euthanize the mice. All animals were euthanized at the end of the experiments and no untimely euthanasia was needed due to worsening of their health status (*e.g*., loss of 20% of original body weight).

### Animals

BALB/cOlaHsd females (ENVIGO, UK) were housed in the Centre for Experimental Biomodels, First Faculty of Medicine (Charles University, Prague); they were used for experiments examining the presence of Aβ in the infected CNS. Heterozygous B6;C3-Tg(APPswe,PSEN1dE9)85Dbo/Mmjax mice (Jackson Laboratory, Farmington, CT, USA; further referred to as “APP/PS1”) and wild type (WT) littermates were bred and genotyped at the Institute of Molecular Genetics (Czech Academy of Sciences, Prague) and housed at the Faculty of Science (Charles University, Prague). APP/PS1 mice overproduce Aβ [19], so they were used to monitor the effect of Aβ on the course of neurotoxocarosis. All mice were caged in groups (4–6 animal per cage) and provided access to food and water *ad libitum*, unless an infection was performed as described below. The mice were given regular care and enrichment (paper shelters, wooden sticks, nesting material) with daily checks of their health status. Allocation of animals to experimental groups was made randomly by a blinded animal technician. The persons performing the experiments were blinded to the experimental group (control, infected) or animal genotype (WT, APP/PS1).

### Culture of *Toxocara canis* larvae

Adult females of *T. canis* were obtained from naturally infected dogs from dog shelters following anthelmintic treatment. Female worms were collected, and *Toxocara* eggs were isolated by sieving female internal organs through a tea strainer and dissolving redundant tissue with 0.2 N H_2_SO_4_ [5]. Hatching of L3 larvae was performed by modified protocols of Fairbairn [17] and De Savigny *et al*. [14] using glass beads to disrupt the eggshell mechanically. Then, vital larvae were separated using Baermann’s apparatus with a 40 μm cell strainer (Corning, Corning, NY, USA). Hatched L3 larvae were placed in cultivation flasks in serum-free RPMI-1640 (Sigma-Aldrich, St. Louis, MO, USA) supplemented by 100 IU/mL penicillin, 250 μg/mL streptomycin, 0.1 IU/mL L-glutamine and 12 mM HEPES. They were used for infection of mice or *in vitro* experiments no later than 30 weeks after hatching. The culture medium containing *Toxocara* excretory-secretory (TES) antigen produced by L3 larvae was collected at weekly intervals according to Novák *et al*. [34] and further used as antigens in ELISA tests.

### *In vivo* experimental design

**Figure 1:**
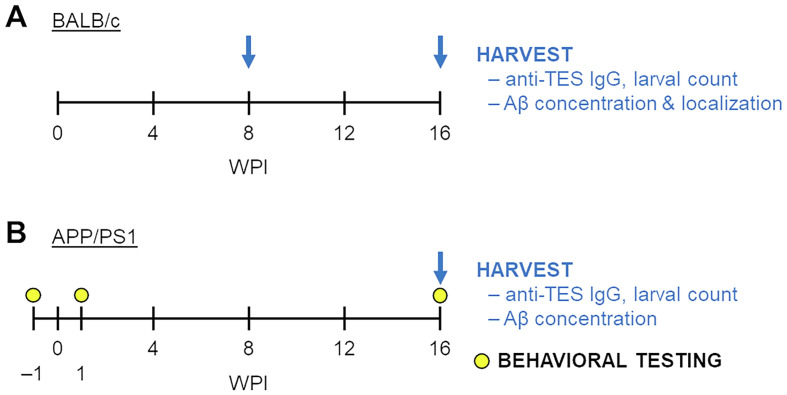
Experimental design of *in vivo* experiments. (A) BALB/c mice were infected with 1,000 L3 larvae and harvested for further analyses 8 and 16 weeks post infection (WPI). (B) APP/PS1 mice were behaviorally tested –1, 1 and 16 WPI, when they were harvested for further analyses.

*In vivo* experiments were conducted to examine if (a) infection with *T. canis* affects Aβ levels in the CNS of BALB/c mice (*n* = 5–7 per group) or facilitates AD-like behavioral changes in APP/PS1 mice (*n* = 3–6 per group), and if (b) Aβ overproduction affects the course of *T. canis* infection in APP/PS1 mice (*n* = 7–11 per group). The infected and control mice were continuously monitored and terminally harvested 8 or 16 WPI. Blood and CNS were collected for downstream analyses as described below. The actual number of animals was decided based on preliminary experiments and is indicated in the figures. The study design is summarized in Figure 1.

### Infection of mice

Infection of 8-week-old female mice was performed similarly to Novák *et al.* [34]. Briefly, 1 day before infection, mice were denied water to boost their subsequent fluid intake. On the day of infection, L3 larvae were washed with phosphate-buffered saline (PBS) and distributed in glass tubes (1,000 L3 in 1 mL of PBS per mouse). After 4 h, the tubes were checked and refilled if necessary to ensure intake of the entire infection dose. The control group received only PBS. The day after the infection, mice were weighed and returned to cages.

### Behavioral tests

APP/PS1 and control WT female mice were behaviorally tested repeatedly at three time points: T1 (1 week before the infection by *T. canis*), T2 (1 WPI), and T3 (16 WPI). This design was adopted due to limited access to APP/PS1 animals. The absence of uninfected controls (both WT and APP/PS1) did not allow us to assess the effect of the infection on the behavioral outcome. However, we were still able to evaluate the effect of the genotype linked to increased Aβ production, which corresponds to the study objectives. All mice underwent three behavioral tests in the following order.

#### Elevated plus maze (EPM)

The plus-shaped apparatus made from white plastic consisted of two opposite open arms and two closed arms (5 × 30 cm; 15 cm high walls) and was elevated 43 cm above the floor. Illumination ranged between ~130 lux (closed arms) and ~320 lux (open arms). The mouse was placed in the central part of the apparatus facing an open arm and left undisturbed for 5 min. The frequency of open arm visits as a measure of anxiety and total walked distance as a measure of locomotor activity were assessed.

#### Open field (OF)

The mouse was placed in the center of the square, white-laminated chipboard arena (50 × 50 cm) with opaque walls (40 cm high) and was left to freely explore for 10 min. The arena was illuminated from ~205 lux (walls) to ~270 lux (center). Locomotor activity (measured by total walked distance) and anxiety (measured by time spent in the center of the arena) were assessed.

#### Y-maze

The apparatus consisted of three identical arms labeled A, B, and C (6 × 35 cm; 20 cm high walls; illuminated ~ 150 lux) made from white plastic. The mouse was placed at the end of the A arm facing the center and was left free to explore the maze for 8 min. Spontaneous alternation (in %) was calculated as the number of correct triads of arm entries (ABC, BCA, CAB, ACB, CBA, BAC) divided by the number of all triads. Spontaneous alternation is a measure of working memory as it is assumed that mice prefer to alternate between the visited arms if they remember past choices. Activity was measured as total arm visits.

The apparatuses were always cleaned with a water-ethanol mixture between individual sessions and dried with paper cloths. All three tests were performed during the same day in the light phase of the daily cycle of the animals. Mice behavior was recorded by an overhead camera (Logitech C920) using Logitech Webcam software. Data were analyzed offline automatically in EthoVision software (Noldus Information Technology, Wageningen, Netherlands; EPM and OF), or manually (Y-maze).

### Sample harvest

Mice were euthanized, blood for serum samples was collected, and transcardial perfusion with heparinized PBS (“unfixed” samples) or PBS and 4% formaldehyde (“fixed” samples) was performed. Lastly, intact brains were extracted. The brains of “unfixed” mice were used for larval burden examination and Aβ concentration measurement (BALB/c and APP/PS1 mice); the brains of “fixed” mice were used for immunofluorescent staining of Aβ (BALB/c mice).

### Serum preparation and anti-*Toxocara* ES IgG ELISA

Clotted blood was centrifuged (1,500 × *g*, 10 min), and collected sera were stored at −80 °C. Levels of specific anti-TES IgG antibodies were measured by ELISA as already described [34].

### Parasite burden

The brains of “unfixed” mice were sliced in half, and one hemisphere, half of the cerebellum, and the brain stem were individually weighed and placed into PBS at 4 °C overnight. Small parts of neural tissue were then compressed between two microscopic slides, and larvae were counted under the light microscope.

### Neural tissue Aβ ELISA

Amyloid beta 42 Mouse ELISA Kits (Invitrogen, Waltham, MA, USA) were used to measure Aβ concentration in the other half of “unfixed” brains. The tissue was homogenized by sonication in 5 M guanidine-HCl/50 mM Tris (pH 8.0), and the soluble fraction was further processed and analyzed according to the manufacturer’s instructions.

### Immunohistochemical staining

The “fixed” samples of mouse CNS were prepared and processed as previously described by Macháček *et al*. [32]. Cryosections (10 μm) were incubated overnight with primary antibodies (polyclonal rabbit anti-mouse Aβ, Rockland; 1:1,000). Anti-rabbit goat secondary antibodies with a fluorophore (Alexa Fluor^®^ 488/594, Cell Signaling Technology, 1:1,000) were subsequently allowed to bind for one hour. Lastly, the slides were mounted in VectaShield with DAPI (VectorLabs, Newark, CA, USA) and observed under the fluorescence microscope (BX 51, Olympus).

### *In vitro* experiments and larval viability

Thirty *T. canis* L3 larvae in the cultivation medium were placed into individual wells of the 96-well plate and were treated with recombinant mouse Aβ_1-42_ (Sigma-Aldrich; dissolved in ddH_2_O) in final concentrations of 5, 50, or 125 μg/mL. The treatment lasted 2 or 7 days, and 12 h before the end, a vital dye fluorescein diacetate (FDA) (Sigma-Aldrich, 0.4 μg/mL) was added. Propidium iodide (PI) (Sigma-Aldrich, 20 μg/mL) was employed as a non-vital dye. For easier observation, the larvae were immobilized by 0.4% formaldehyde. Larvae viability was assessed under the fluorescence microscope: FDA+ larvae were considered viable, PI+ larvae were considered dead. Additionally, we monitored the larval morphology: viable larvae were curled and intact, while dead larvae were characteristically taut and/or visibly damaged.

### Scanning electron microscopy

L3 larvae were fixed for one hour using 0.1 M cacodylate buffer solution (CBS) containing 2.5% glutaraldehyde and 1% formaldehyde. Then, triple 5-minute CBS rinsing followed. One-hour post-fixation employing 1% osmium tetroxide (in CBS) was again followed with three times CBS rinsing. The larvae were subsequently dehydrated in the increasing ethanol series (30%, 50%, 75%, 85%, 95%, 2 × 100%; 5 min each). Critical point drying was performed in acetone by Leica EM CPD300; the larvae were then mounted and coated with a 3 nm layer of Pt using Leica EM ACE600, and later 3 nm Au using Bal-Tec SCD 050. Imaging was performed on a JEOL 6380 LV scanning electron microscope.

### Statistical analysis and data visualization

All quantitative data were statistically analyzed and visualized in GraphPad Prism 10 (Dotmatics), showing individual data along with group medians. The data were checked for normality (Shapiro-Wilk test, QQ-plot) and analyzed as specified in the figure legends (1- or 2-way ANOVA or mixed-effects model followed by *post hoc* tests or Kaplan-Meier curve & Mantel-Cox test for survival). Statistical significance is indicated by asterisks: *p ≤ 0.05, **p ≤ 0.01, ***p ≤ 0.001, ****p ≤ 0.0001.

## Results

### Chronic murine neurotoxocarosis did not elevate Aβ concentrations in the CNS

**Figure 2:**
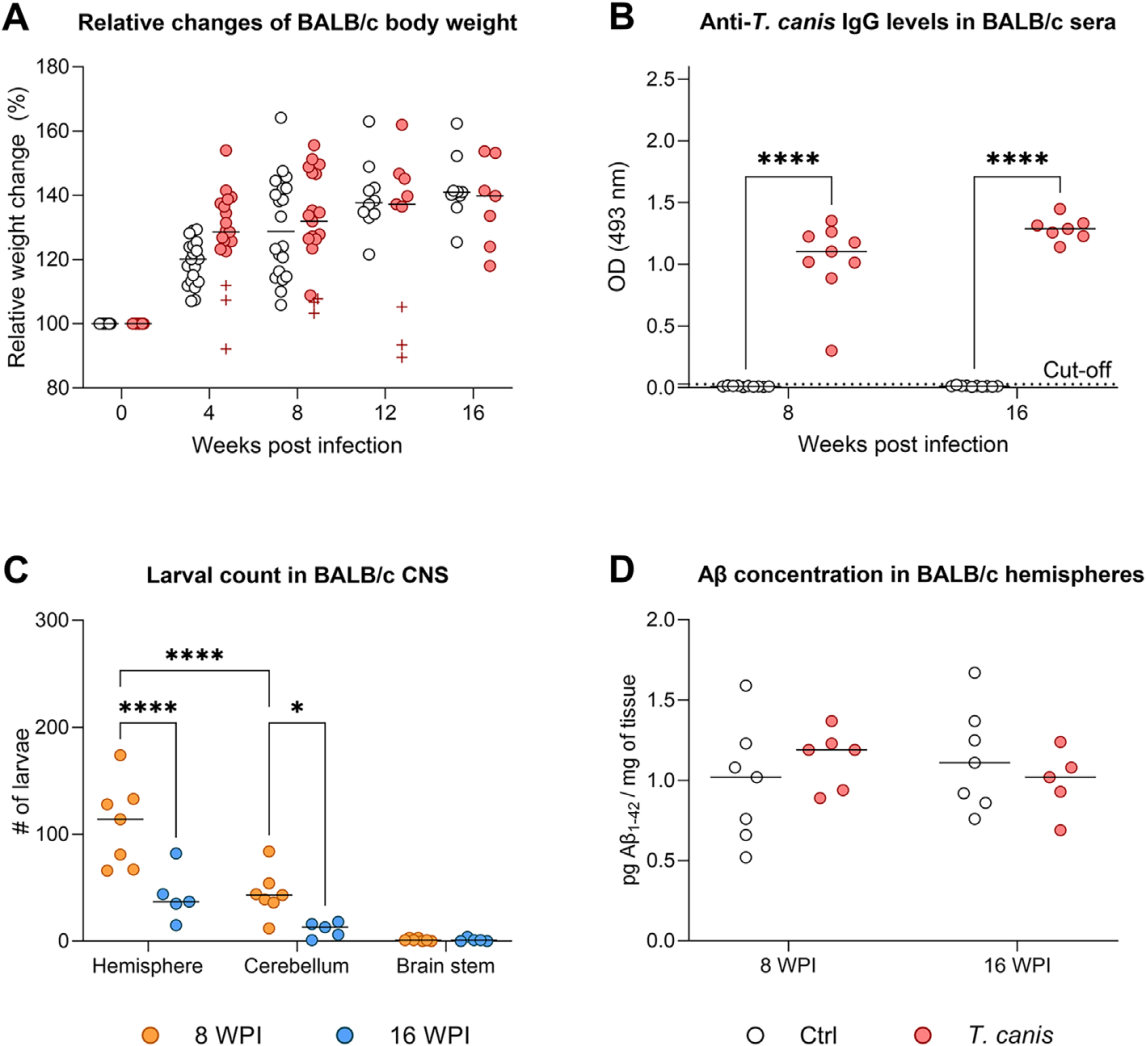
Characterization of chronic neurotoxocarosis in BALB/c murine model. (A) Relative weight changes did not differ between infected and control groups. Crosses (“+”) indicate mice that showed body weight drop; two of them died 13 WPI. The graph contains mice pooled from two experiments (harvest 8 and 16 WPI). (B) Levels of serum anti-*T. canis* IgG antibodies were significantly elevated in infected mice at both time points. (C) *T. canis* larvae accumulated mostly in hemispheres at both time points. Overall, more larvae were found in the group sacrificed 8 WPI. The values show larval burden in half of the freshly harvested organ as the other half was used for Aβ measurement (see next). (D) Chronic neurotoxocarosis did not alter Aβ production in the hemispheres. Individual data are shown along with the group median. For statistical analysis, a mixed-effects model (A) or 2-way ANOVA (B–D) followed by Holm-Šidák’s multiple comparisons test were used. Significant differences among the infected groups are indicated by asterisks (*p ≤ 0.05, ****p ≤ 0.0001). Aβ, amyloid-β; CNS, central nervous system; Ctrl, control (uninfected) group; OD, optical density; WPI, weeks post infection.

Infection of BALB/c mice with *Toxocara canis* did not significantly affect gain in body weight when compared to healthy controls (Fig. 2A). Only one infected mouse died 3 WPI, and three infected mice showed a remarkable drop in body weight after 4 WPI (marked with crosses in Fig. 2A). They also exhibited certain behavioral changes (tumbling motion and body posture, stereotypic movements) and two of them died 13 WPI. However, no significant alteration in probability of survival was noticed between control and infected mice (*p* = 0.07). All infected mice harvested 8 or 16 WPI showed significantly increased serum levels of anti-TES IgG (Fig. 2B). Total larval burden in the CNS was higher in the hemispheres than in the cerebella or brain stems; more larvae were found 8 WPI than 16 WPI (Fig. 2C). However, no changes in Aβ concentration were detected in infected hemispheres either 8 or 16 WPI (Fig. 2D); similar results were obtained for the cerebella (data not shown).

### Aβ was associated with the outer surface and intestine of *T. canis* larvae in the CNS

**Figure 3:**
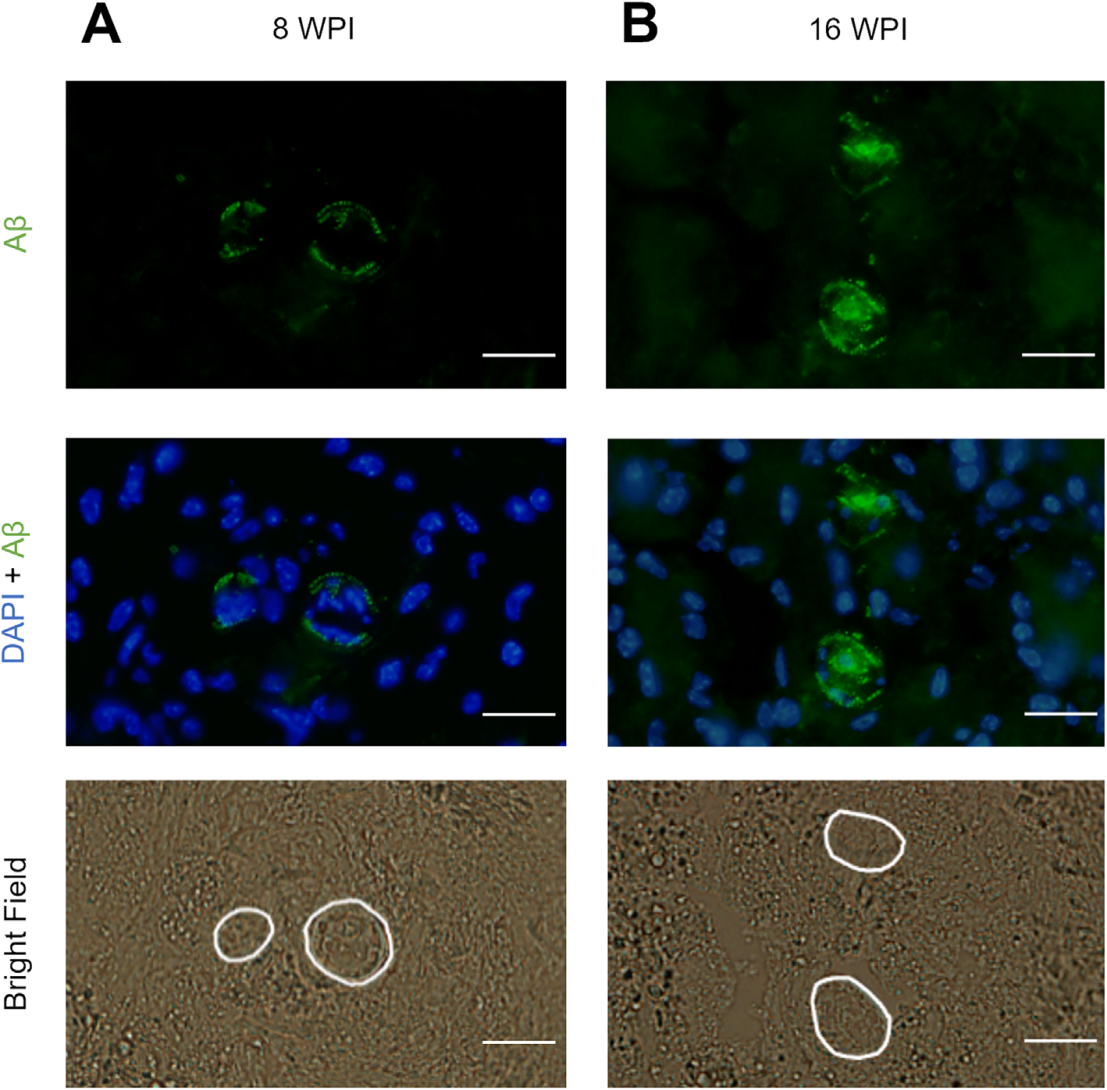
Immunohistochemical localization of Aβ associated with *T. canis* larvae in BALB/c CNS. Signal for Aβ was located only on the surface and in the intestine of found larvae in 8 WPI (A) and 16 WPI (B) as well. The position of sectioned larvae is depicted by white lines in the bright field photos. Beyond the round shape in the cross section, the larvae can be distinguished by smaller nuclei packed more densely within the parasite tissues. Three mice per time point and at least three larvae-positive slides per mouse were analyzed. Aβ, amyloid-β; WPI, weeks post infection. Scale bars: 20 μm.

Although increased Aβ concentration was not observed in the infected CNS, an attempt was made to localize Aβ in the nervous tissue to assess whether Aβ is somehow associated with the migrating larvae. This could remain unnoticed by the ELISA quantification, but could still indicate relevant host-parasite interplay. Hence, Aβ immunofluorescence staining was performed on control and infected animals 8 and 16 WPI. In infected brains, the only observed signal for Aβ was on the larval surface and likely in the intestine (Fig. 3). No signs of Aβ plaques were detected throughout the neural tissues. The specificity of staining was monitored by incubation of larvae-positive slides with negative rabbit serum as well as without either primary or secondary antibodies (not shown). Uninfected mice were also investigated for Aβ signal in CNS in both time points, but there were no noticeable sources of signal (not shown).

### Recombinant Aβ_1-42_ did not alter the viability of *T. canis* larvae *in vitro*

**Figure 4:**
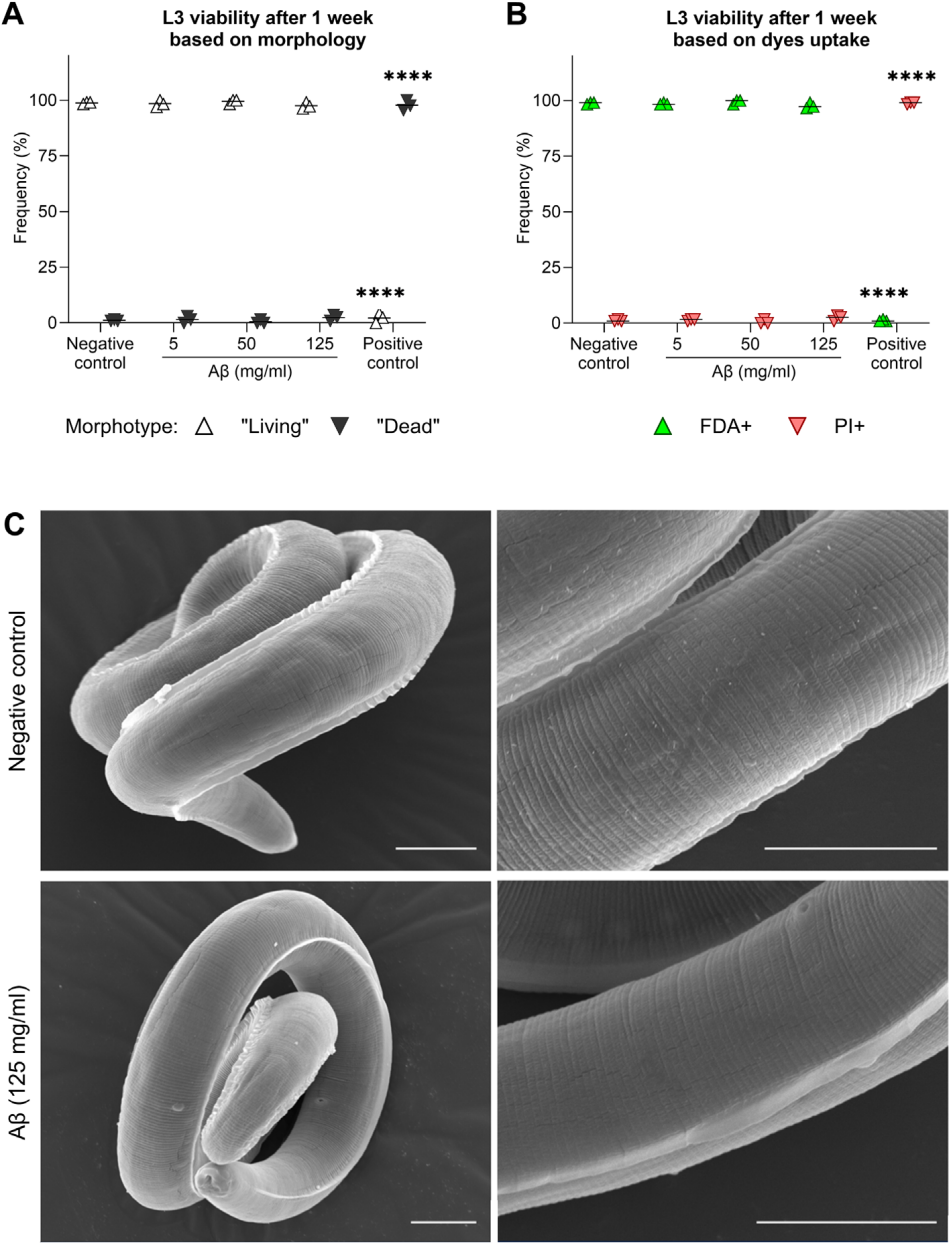
Viability assessment of *T. canis* larvae after *in vitro* treatment with recombinant Aβ_1-42_. After one week of treatment, neither morphology (A) or staining with vital (fluorescein diacetate; FDA) and non-vital (propidium iodide; PI) dyes (B) showed alterations in larval viability. Moreover, the 7-day treatment did not disrupt the surface of larvae as revealed by scanning electron microscopy (C). For statistical analysis, 1-way ANOVA followed by Dunnett’s multiple comparisons test were used to compare the treated groups to the respective negative control. Significant differences are indicated by asterisks (****p ≤ 0.0001). Scale bars: 10 μm.

As Aβ was associated with both the outer and inner surfaces of *T. canis* larvae in the brain, Aβ capacity to injure them was tested *in vitro*. However, incubation with physiological Aβ concentrations did not reveal any changes in parasite morphology (Fig. 4A) or staining by vital (FDA) and non-vital (PI) dyes (Fig. 4B). This suggests the absence of Aβ larvicidal capacity both after 2 and 7 days of treatment. This conclusion was also confirmed by observation of the treated larvae by scanning electron microscopy, which did not indicate any surface changes or damage (Fig. 4C).

### Overproduction of Aβ in APP/PS1 mice did not control *T. canis* neuroinfection

**Figure 5:**
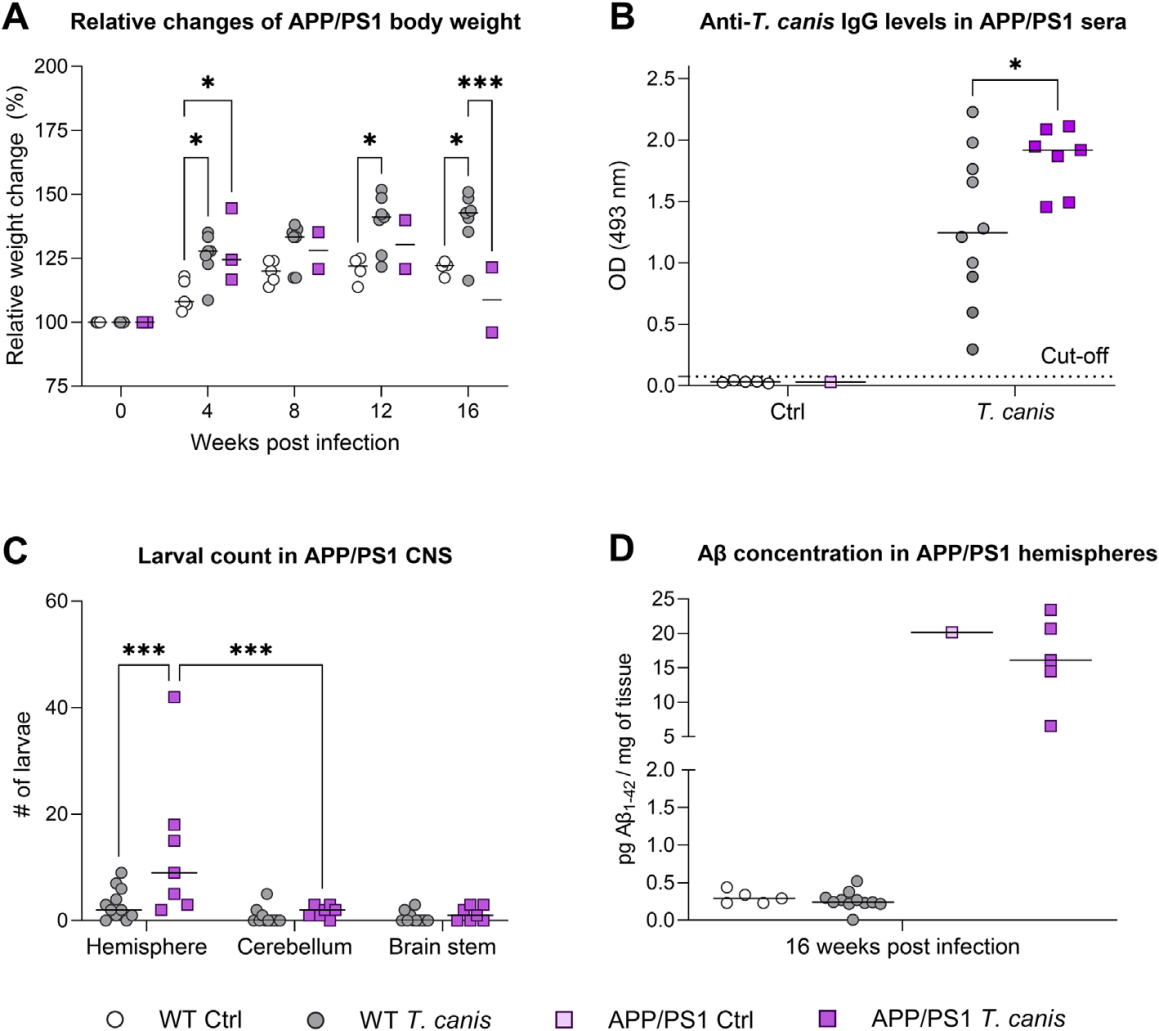
Characterization of chronic neurotoxocarosis in APP/PS1 murine model. (A) Relative weight changes differ between infected and control groups and also between infected WT and APP/PS1 mice at 16 WPI. The graph contains only data from mice that did not undergo behavioral testing. (B) Levels of serum anti-*T. canis* IgG antibodies were elevated both in infected WT and APP/PS1 mice, the latter showing even significantly higher values. (C) *T. canis* larvae mostly preferred the hemispheres in both groups. Interestingly, more larvae were found in the APP/PS1 group. The values show larval burden in half of the freshly harvested organ as the other half was used for Aβ measurement (see next). (D) Chronic neurotoxocarosis did not alter Aβ production in the hemispheres, higher levels of Aβ were observed in the APP/PS1 groups as expected. Individual data are shown along with the group median. For statistical analysis, a mixed-effects model (A) or 2-way ANOVA (B–D) followed by Holm-Šidák’s multiple comparisons test were used. Significant differences among the infected groups are indicated by asterisks (*p ≤ 0.05, ***p ≤ 0.001). Aβ, amyloid-β; CNS, central nervous system; Ctrl, control (uninfected) group; OD, optical density; WPI, weeks post infection.

To evaluate the effect of increased Aβ concentration on *T. canis* larvae *in vivo*, APP/PS1 mice overproducing Aβ were infected and harvested 16 WPI. No significant changes in weight gain were detected between infected WT and APP/PS1 mice, except for a drop in APP/PS1 mice at 16 WPI (Fig. 5A). Somewhat decreased probability of survival was noticed in APP/PS1 mice as two mice died during the infection (3 and 7 WPI, *p* = 0.05). Interestingly, infected APP/PS1 individuals had higher serum levels of anti-TES IgG (Fig. 5B). Compared to BALB/c mice, the total larval burden was generally lower in WT and APP/PS1 animals. Of note, significantly more larvae were found in the hemispheres of APP/PS1 mice than in WT littermates (Fig. 5C). However, the infection did not markedly change Aβ concentration in the CNS (Fig. 5D).

### Behavioral tests

#### Elevated plus maze

**Figure 6:**
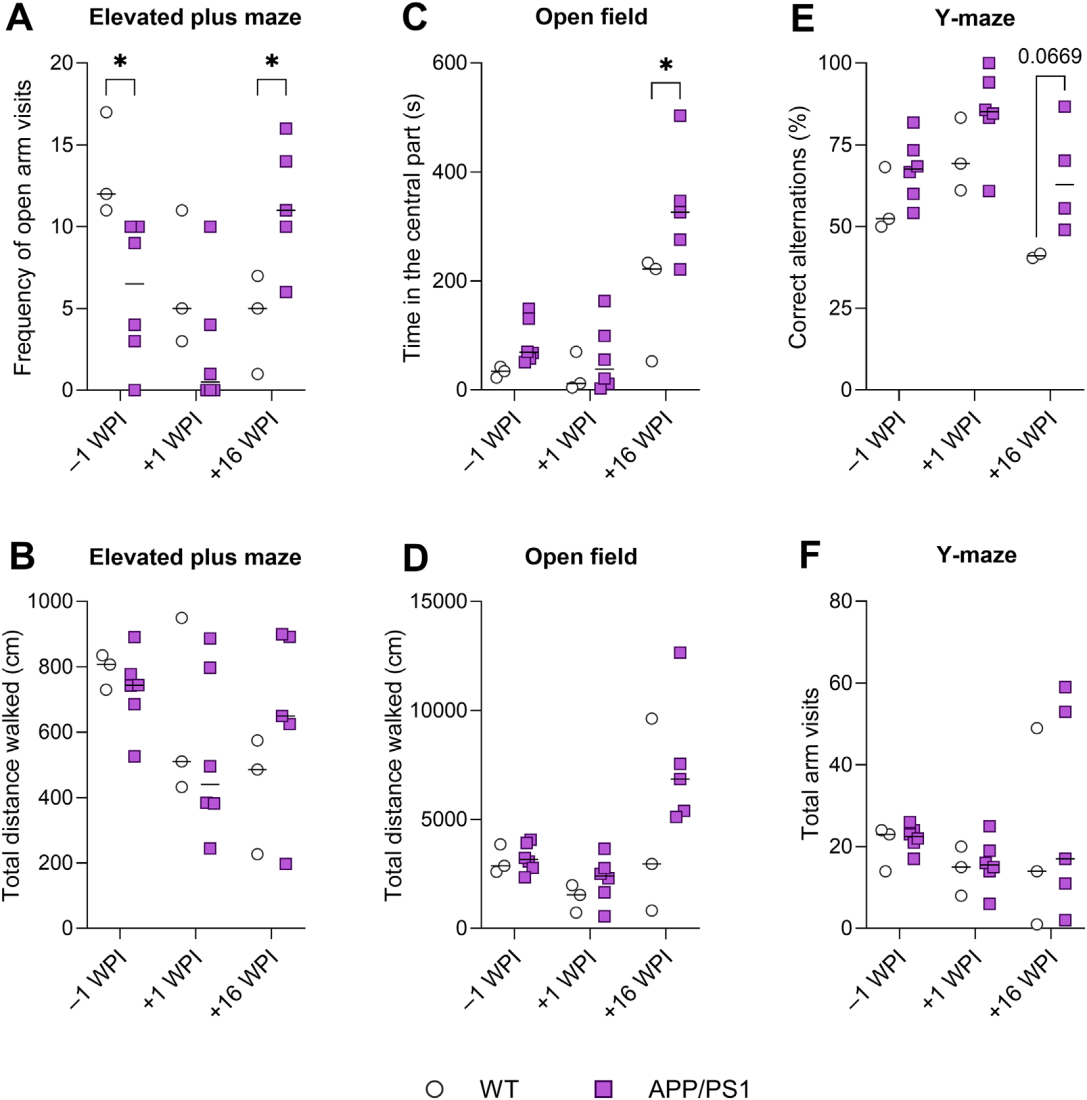
Influence of neurotoxocarosis on behavior of APP/PS1 mice. (A) Frequency of open arm visits was lower in APP/PS1 compared to WT mice –1 WPI, but notably increased 16 WPI. (B) Total distance walked was altered by neither genotype nor time point. (C) APP/PS1 mice spent more time at the center of the open field compared to WT across all time points; this parameter was notably increased 16 WPI in both groups. (D) Total distance walked in the open field was comparable between WT and APP/PS1, with a dramatic increase 16 WPI seen in both groups. (E) Spontaneous alternation was not affected by genotype but differed across time points. (F) Total arm visits were affected by neither time point nor genotype. Individual data are shown along with the group median. A mixed-effects model was used along with Holm-Šidák’s multiple comparisons test to detect differences at particular time points. Significant differences among WT and APP/PS1 mice are indicated by asterisks (*p ≤ 0.05). If the animal fell from the apparatus (EPM) or was not moving (Y-maze), it was excluded from the data sets, which explains lower n indicated in some cases.

APP/PS1 mice and their WT counterparts differed in their frequency of open arm visits (Fig. 6A), as evidenced by a significant interaction between the factors of time point and genotype (*p* = 0.0045). Prior to and a week after infection, APP/PS1 mice entered open arms rather less often, suggesting more anxious behavior, while 16 WPI, they visited open arms much more often than WT mice, suggesting lower anxiety. Total distance walked was affected by neither genotype nor time point, showing normal locomotor abilities (Fig. 6B). However, we observed frequent stereotypic behaviors and postural problems, including falls from the open arms, in both groups at 16 WPI.

#### Open field

APP/PS1 mice spent more time in the central part of the apparatus, the difference was statistically significant at 16 WPI (Fig. 6C), suggesting decreased anxiety in APP/PS1 animals. Interestingly, the time spent in the center notably increased in both groups 16 WPI. Locomotor activity was not affected by the genotype but changed between time points, with a mild decrease at 1 WPI and a dramatic increase at 16 WPI (Fig. 6D). The latter might be attributed to frequent stereotypic behavior (circling) in chronically infected mice.

#### Y-maze

Spontaneous alternation changed between time points, but the effect of genotype was not significant (Fig. 6E). No significant effects were found in the total number of arm visits (Fig. 6F). This suggests that both working memory and locomotor activity were not affected by genotype.

An overview of the behavioral data suggests that prior to and immediately after infection, APP/PS1 exhibited normal locomotor activity. They were more anxious in the elevated plus maze, but less anxious in the open field. This difference may reflect a specific response to anxiogenic stimuli particular to a given task: fear of heights and avoidance of open spaces, respectively. At 16 WPI, the behavior of the animals in both groups was severely affected by the infection, with stereotypic movements and disruption of normal behavioral patterns. APP/PS1 mice exhibited lower anxiety (more frequent open arm visits, and more time spent in the central part). However, the small sample size at 16 WPI makes any conclusions tentative.

## Discussion

AD has been recognized for over a century, yet its triggers remain largely unknown, limiting prevention and treatment strategies. While pathological processes such as Aβ overproduction and tau hyperphosphorylation are well documented [29], their initiating factors remain unclear. Here, we used a mouse model of chronic neurotoxocarosis to investigate the emerging hypothesis that infections contribute to AD pathogenesis via Aβ’s antimicrobial properties.

*Toxocara canis* has been proposed as a possible AD-triggering infectious agent, especially due to its global prevalence and persistence in the host CNS [18]. Supporting this view, Chou *et al*. [10] recorded by a semi-quantitative Western blot the increased signal of Aβ in the hippocampus of infected mice 8–20 WPI. However, our quantitative study, using ELISA to detect total soluble Aβ, shows no elevated Aβ concentration in the CNS (neither hemispheres nor cerebella) of chronically infected mice – despite the presence of considerable larval burden, particularly in the hemispheres. Moreover, no Aβ deposits were seen throughout the infected CNS, including the hippocampus, when examined by immunohistochemistry. This corroborates the data ascribing the increased levels of AβPP, the Aβ precursor detected in *T. canis* infected brains, to axonal injury, not AD-like pathology [21, 40]. This type of interpretation is further supported by the downregulation of the “Alzheimer disease-amyloid secretase pathway” noted on 16 WPI [45]. Collectively, our data seem to contradict the hypothesis that chronic neurotoxocarosis would facilitate Aβ accumulation, at least within 16 WPI, even in the mouse model naturally overproducing Aβ.

However, it must be acknowledged that our study tracks the effects of *T. canis* infection only up to 16 WPI, which may not be sufficient to capture delayed or progressive AD-like pathology in mice. This follow-up period could limit our ability to detect potential long-term effects of the infection. Additionally, the absence of age-matched healthy controls at 16 WPI and the small sample sizes in the behavioral tests limit the interpretation and statistical power to detect possible subtle effects. Therefore, future studies with extended timeframes and larger sample sizes are needed to fully assess the long-term impact of *T. canis* infection.

Interestingly, while Aβ levels did not increase in the brain tissue, immunofluorescence staining revealed Aβ localized on the surface and within the intestine of migrating *T. canis* larvae. A similar phenomenon of Aβ binding onto the pathogen surface has been observed in viruses, bacteria, and yeasts [16, 27, 38, 39]. This could be related to the proposed role of Aβ in brain innate immunity, which attempts to sequester or injure the invading pathogens [24, 44]. However, our *in vitro* experiments do not support the view that Aβ has anthelmintic activities as both the morphology and viability of Aβ-treated *T. canis* larvae remained unchanged, even after 7-day Aβ treatment. Additionally, the larval burden was not decreased in APP/PS1 mice that naturally overproduce Aβ in the CNS [19], which we also confirmed by ELISA. The lack of antiparasitic activity can be explained by the complex, multi-layered nature of the nematode cuticle, which provides robust resistance against antimicrobial peptides [6, 11]. Additionally, it cannot be excluded that TES containing peptidases [12] help the larvae to remove the surface-bound Aβ. Also, the brain immune milieu is shifted to anti-inflammatory response in infected mice [46], which might decrease Aβ production. As for the Aβ detected in the larval intestine, we speculate that it originates from active feeding of the parasites on the nervous tissue, which was also demonstrated, *e.g*., in the neurotropic schistosome *Trichobilharzia regenti* [31]. Overall, our data show that Aβ is not detrimental to the parasite and that Aβ might not function as a defense mechanism against helminth neuroinfection.

## Conclusion

Altogether, our robust experimental data show that *T. canis* infection does not trigger AD-like pathology in mice and Aβ does not act as an antiparasitic agent. The absence of Aβ elevation in response to *T. canis* infection challenges the notion that parasitic infections can directly exacerbate AD-like pathology through Aβ accumulation [18]. Additionally, this aligns with the recent epidemiologic report showing no strong association between *Toxocara* exposure and AD in humans [25]. Likewise, we found no experimental evidence supporting the hypothesis that Aβ acts as an antimicrobial agent limiting nematode neuroinfections [44]. By demonstrating that *T. canis* is likely not significantly involved in disease etiology in the mouse model, we do not however disprove the AD infectious hypothesis as a whole.
